# Letter to the Editor: Allert et al. “The influence of epidural anesthesia in pregnancies with scheduled vaginal breech delivery at term: a hospital-based retrospective analysis” in the Archives of Gynecology and Obstetrics (published online: 20th November 2023)

**DOI:** 10.1007/s00404-024-07517-y

**Published:** 2024-04-30

**Authors:** Massimiliano Lia, Holger Stepan

**Affiliations:** Department of Obstetrics, University Hospital of Leipzig, 04103 Leipzig, Germany

Dear Editor,

The article by Allert et al. [1] describes the effect of epidural anesthesia in vaginal breech birth. We thank the authors for their efforts in making this analysis on a cohort of 1413 vaginally intended breech births. However, we would like to express some reflections regarding their statistical analysis, which the reader should be aware of when interpreting the results of this study:

1. The authors’ conclusion that “among the patients who delivered vaginally epidural anesthesia was associated with a higher chance of assisted vaginal delivery “ should be expanded upon. Specifically, the group of women with epidural anesthesia had a higher percentage of nulliparous women (64.7%) than those without epidural anesthesia (37.5%). Additionally, we know from another FRABAT-study that nulliparous women have a significantly higher rate of manual assistance than multiparous women [2]. Consequently, the higher rate of manual assistance in women with epidural anesthesia could be explained by the higher percentage of nulliparous women in this group.
  To support this theory, we performed an additional analysis on a cohort of nulliparous women published by us [3] who accomplished vaginal breech birth. Possible predictors for assisted vaginal delivery (including epidural anesthesia) were used to generate prediction models with every possible combination of these predictors. The resulting 4250 models were characterized in terms of their ability to predict assisted vaginal delivery and the 100 best performing models were analyzed further. We then examined how often the various predictors appeared in these 100 best prediction models. Compared to other more important predictors, epidural anesthesia was rarely chosen for those models with good prediction for assisted manual delivery in vaginal breech birth (Fig. 1).
  In summary, it would be more informative for the reader if the effect of epidural anesthesia on manual assistance would have been adjusted for parity (i.e. adjusted odds ratio).
2. The authors’ conclusion that “in vaginal deliveries, the duration of the labor was significantly longer in deliveries with an epidural anesthesia” should be expanded upon. The longer duration of labor with epidural anesthesia could be (partly) explained by the observation that nulliparous women (who usually have longer births) are more likely to receive this kind of pain relief. Consequently, it would be more informative for the reader if the effect of epidural anesthesia on birth duration would have been adjusted for parity.
3. The authors’ conclusion that “patients receiving an epidural anesthesia had a higher probability for cesarean delivery … but when only primiparous women were analysed, cesarean delivery rates were not significantly different “ should be discussed more profoundly. This result stands in contrast to numerous studies published previously, including a FRABAT-study where epidural anesthesia was indeed associated with a higher rate of cesarean section in nulliparous women [4]. We think that this result by Allert et al. should be put into perspective, discussed and compared to those of other studies (including their own) published previously.
4. The authors’ conclusion that “PREMODA scores were consistently not different between deliveries with and without epidural anesthesia” should be discussed in relation to their finding reported in Table  3. Here, the authors report a significant (*p* = 0.0414) difference of the PREMODA score between nulliparous women with and without epidural anesthesia.

**Fig. 1:**
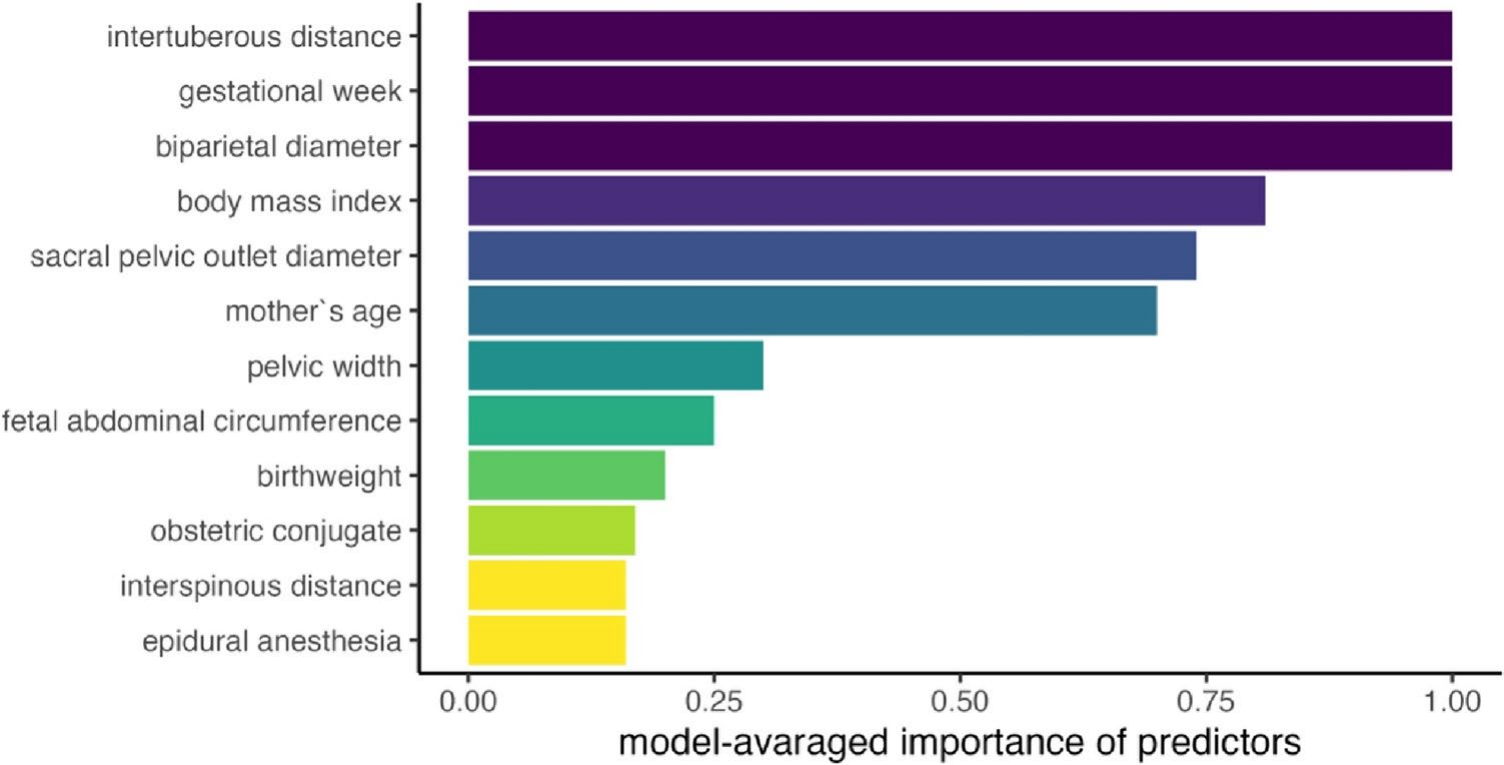
Relative importance of various predictors based on the analysis of the 100 models with the best prediction for assisted vaginal breech delivery in a cohort of 268 nulliparous women of the University Hospital of Leipzig

We applaud the authors for providing valuable evidence on the effects of epidural anesthesia in breech delivery. However, the readers would have profited from a more detailed interpretation and discussion of the results in this study.

## Data Availability

The data used for the presented analysis are available from the corresponding author upon reasonable request.
